# A case report on peripheral cemento-ossifying fibroma in South India

**DOI:** 10.6026/973206300200771

**Published:** 2024-07-31

**Authors:** Pavithra Gopalakrishnan, Vijayalakshmi Rajaram, Lakshmi Priya Kannan, Anitha Logaranjani, Jaideep Mahendra

**Affiliations:** 1 Department of Periodontics, Meenakshi Ammal Dental College and Hospital, Maduravoyal, Chennai, Tamil Nadu, India

**Keywords:** Gingival enlargement, *peripheral cemento-ossifying fibromas*, electrocautery

## Abstract

Gingival enlargement is a common manifestation of gingival diseases and it is characterized by increase in size of the gingiva. A 20-year-old male reported with the primary complaint of swelling in lower front teeth region for the past 7 month. Patient noticed the swelling to be small when it started and gradually increased to attain the present size. There was no contributing history of bleeding or pain. Excision of the lesion was done using electrocautery followed by histopathological analysis. The challenge for clinicians lies in accurately diagnosing the underlying cause due to the diverse presentations of these conditions in this case report.

## Background:

Gingival enlargement also known as gingival overgrowth or hypertrophy is a common manifestation of gingival diseases and it is characterized by increase in size of the gingiva. Gingival enlargement can result from various factors including inflammation, medications, systemic diseases, neoplastic, false enlargement and genetic predispositions.[1] Gingival enlargement alters the cell size, cell proliferation, gingival vasculature and the extracellular matrix. These changes can impact the aesthetics, mastication, speech, and oral hygiene practices.[2] The challenge for clinicians lies in accurately diagnosing the underlying cause due to the diverse presentations of these conditions. Accurate diagnosis involves a comprehensive approach considering clinical, radiographic, histopathological and patient-related factors.

## Case report:

**Figure 1:**
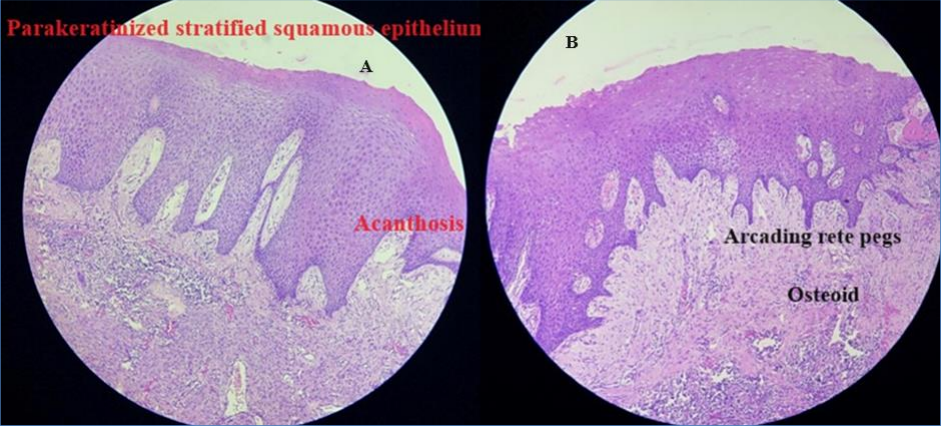
Histological examination

A 20-year-old male reported to the Department of Periodontics, Meenakshi Ammal Dental College, Chennai with the chief complaint of swelling in lower front teeth region, for the past 7 months. Patient gives history of accidental hit during football practice following which the patient developed swelling in lower anterior. Patient noticed the swelling to be small when it started, and gradually increased to attain the present size. There was no contributing history of bleeding or pain. The patient was systemically healthy. Intra-oral examination revealed a gingival overgrowth in relation to 31, 32 and 41 with no color change, approximately measuring 1x1.1cm in size, pedunculated, surface which was smooth, non-ulcerated and with a broad attachment base. The consistency of soft tissue growth was firm and fibrotic. Teeth associated with it showed plaque and calculus with grade I mobility. Radiographic examination using intra-oral periapical radiograph irt 31,32,41,42 revealed crestal bone loss. Based on the above findings, a provisional diagnosis of traumatic fibroma or irritational fibroma was made. The differential diagnosis included pyogenic granuloma and giant cell granuloma. Routine blood investigation (RBC, WBC, platelet, haemoglobin, bleeding time and clotting time) was advised, which was found to be within the normal limit. One week following thorough oral prophylaxis, surgical excision and biopsy was planned on the basis of the clinical and radiographic examinations. Biopsy serves as a valuable tool in oral diagnosis. This procedure enables the confirmation or rejection of a diagnosis, allowing for the determination of the nature and characteristics of the lesion and ultimately contributing to the establishment of a definitive diagnosis.[3] Surgical excision was done using electrocautery which involves transmission of radio frequency or high-frequency electrical current through tissue to achieve a specific clinical outcome. This energy is utilized to either cut or coagulate the tissue. When high-frequency electrical current is applied, the tissue essentially vaporizes as the electrode passes through it, and capillaries along the incision site are sealed as the tissue contracts.[4] After administration of local anesthesia, excision of the lesion was done using electrocautery and send for biopsy. The excised tissue was stored in *10% neutral buffered formalin* and sent to the Department of Oral Pathology for histopathological examination. Area of then debrided and periodontal dressing (non-eugenol Coe-pak) was placed. Paracetamol 500mg for 3days was prescribed to the patient to control postoperative discomfort. No antibiotics were prescribed. Post-operative instructions were given. The patient was recalled after 1 week for removal of dressing and post-operative follow-up. Routine *haematoxylin* and *eosin* stain was used for the biopsied tissue. Histopathological report revealed, parakeratinized stratified squamous epithelium exhibiting acanthosis and arcading rete hyperplasia admixed with areas of ulceration. Beneath the ulceration, numerous sprouting endothelial lined engorged dilated capillaries resembling granulation tissue and mixed inflammatory cell infiltrate predominantly plasma cells, lymphocytes and neutrophils along with foamy macrophages are evident. The adjacent connective tissue is cellular exhibiting plump fibroblasts, basophilic globular masses resembling cementum and eosinophilic areas resembling osteoid (Figure 1). Based on the above histopathological report, a final diagnosis of peripheral cemento-ossifying fibroma was made. The patient was recalled every third month for maintenance therapy and to check for oral hygiene status. Oral hygiene instructions were reinforced. The recall was done for 1year.

## Discussion:

*Peripheral cemento-ossifying fibromas* (PCOF) are benign non-neoplastic lesions that typically originate from the periodontal ligament or the gingival soft tissue overlying the alveolar process of the jaws. They often appear as nodular masses, either pedunculated (attached by a stalk) or sessile (broad-based) and can vary in color from red to pink. The lesion's surface is frequently ulcerated, and mild crestal bone loss is observed as an early clinical feature. Shepherd initially reported peripheral cemento-ossifying fibroma in the year 1844 as alveolar exostosis. The term "peripheral ossifying fibroma" was later coined by Eversole and Robin in 1972. Peripheral cemento-ossifying fibroma is also called as peripheral cementifying fibroma, calcifying or ossifying fibroid epulis, mineralizing ossifying pyogenic granuloma, peripheral fibroma with calcifications and calcifying fibroblastic granuloma. The central type of ossifying fibroma arises from the endosteum of the bone or the periodontal ligament near the root apex, leading to bone expansion. On the other hand, the peripheral type, as described in our case, occurs on the soft tissue overlying the alveolar process. The exact etiology of ossifying fibromas is not fully understood, but they are often associated with trauma or local irritation, such as fractures or injuries. In our case report, the patient gives history of trauma which could be the reason for peripheral cemento-ossifying fibroma. These fibromas can indeed continue to enlarge if left untreated. [5] Identification of local irritants such as plaque and calculus play a significant role in the etiology and exacerbation of gingival enlargement. Poor oral hygiene can lead to plaque accumulation, which in turn can trigger inflammatory responses and gingival overgrowth. Approximately two-thirds of all cases occur in females, showing a higher predilection in the anterior maxilla. Hormonal influences could be a contributing factor, as evidenced by the higher prevalence of peripheral cemento-ossifying fibroma among females, a rise in occurrence during the second decade, and a decrease in incidence after the third decade. However, in our case a male patient was diagnosed with peripheral cemento-ossifying fibroma in mandibular anteriors. The size of peripheral cemento-ossifying fibroma varies from 0.4 to 4.0 cm, and it is more commonly observed in whites accounting for about 71% [9,10]. Peripheral cemento-ossifying fibroma most frequently occurs in the gingiva, leading to the assertion that these tumors originate from the periodontal ligament. This is attributed to the proximity of the gingiva to the periodontal ligament (PDL) space (Walters *et al.* 2001) [6] and the presence of oxytalan fibers within the mineralized matrix of the same lesion (Buchner *et al.* 1987). [7] When there is gingival injury caused by irritation from a foreign object or subgingival calculus, it results in excessive proliferation of mature fibrous connective tissue. Chronic irritation of the periosteal and periodontal ligament fibers leads to metaplasia of the connective tissue and dystrophic calcification due to irritation of periosteal bone. It is crucial to promptly identify and address such lesions. The treatment modalities include surgical excision using a scalpel, laser, or electrocautery. Surgical excision involves removing the affected periodontal ligament and periosteum. Electrocautery offers several advantages, such as minimal post-surgical pain, the potential to avoid sutures at the biopsy site, minimal intraoperative bleeding, reduced post-operative pain, and excellent healing within one week. The clinical features of peripheral cemento-ossifying fibroma often resemble those of extraosseous lesions, which can lead to a misdiagnosis. Therefore, diagnosing peripheral cemento-ossifying fibroma solely based on clinical aspects can be challenging and misleading. Histopathological examination of the surgical specimen is essential for an accurate diagnosis. In this case, all the classic histopathological features of peripheral cemento-ossifying fibroma were observed. A detailed patient history can often provide clues to the underlying cause of gingival enlargement. Factors such as medication use, systemic conditions, oral hygiene habits, and familial history are essential to consider. Observation on the location, size, color, texture and consistency of the gingival enlargement aids in differential diagnosis for adequate treatment. Effective plaque control through proper oral hygiene measures (brushing, flossing, and professional cleanings) is fundamental in managing and preventing gingival enlargements. Removal of local irritants helps to mitigate inflammation and reduce the risk of further gingival enlargement. In challenging cases where the diagnosis is uncertain or uncommon causes are suspected, biopsy is necessary. Excisional or incisional biopsy allows for histopathologic examination of the tissue, which can provide a definitive diagnosis. Histopathologic examination helps to differentiate between various gingival pathologies, including inflammatory, neoplastic, and genetic conditions. By integrating these diagnostic and management principles, clinicians can effectively assess and treat gingival enlargements, ensuring optimal oral health outcomes for patients. [11]

## Conclusion:

A multidisciplinary approach involving clinical examination, histopathological confirmation and complete surgical excision in managing peripheral cemento-ossifying fibroma, provides a comprehensive understanding of the condition and guides in effective treatment strategies. Regular follow-ups after excision are also crucial to monitor any signs of recurrence and ensure the patient's long-term oral health.
